# A Review on Nano-Based Drug Delivery System for Cancer Chemoimmunotherapy

**DOI:** 10.1007/s40820-020-00482-6

**Published:** 2020-07-05

**Authors:** Weiwei Mu, Qihui Chu, Yongjun Liu, Na Zhang

**Affiliations:** Department of Pharmaceutics, Key Laboratory of Chemical Biology (Ministry of Education), School of Pharmaceutical Sciences, Cheeloo College of Medicine, Shandong University, 44 Wenhuaxi Road, Jinan, 250012 People’s Republic of China

**Keywords:** Cancer therapy, Chemotherapy, Immunotherapy, Chemoimmunotherapy, Nano-based drug delivery system

## Abstract

Although notable progress has been made on novel cancer treatments, the overall survival rate and therapeutic effects are still unsatisfactory for cancer patients. Chemoimmunotherapy, combining chemotherapeutics and immunotherapeutic drugs, has emerged as a promising approach for cancer treatment, with the advantages of cooperating two kinds of treatment mechanism, reducing the dosage of the drug and enhancing therapeutic effect. Moreover, nano-based drug delivery system (NDDS) was applied to encapsulate chemotherapeutic agents and exhibited outstanding properties such as targeted delivery, tumor microenvironment response and site-specific release. Several nanocarriers have been approved in clinical cancer chemotherapy and showed significant improvement in therapeutic efficiency compared with traditional formulations, such as liposomes (Doxil^®^, Lipusu^®^), nanoparticles (Abraxane^®^) and micelles (Genexol-PM^®^). The applications of NDDS to chemoimmunotherapy would be a powerful strategy for future cancer treatment, which could greatly enhance the therapeutic efficacy, reduce the side effects and optimize the clinical outcomes of cancer patients. Herein, the current approaches of cancer immunotherapy and chemoimmunotherapy were discussed, and recent advances of NDDS applied for chemoimmunotherapy were further reviewed.

## Highlights

- The current approaches of cancer immunotherapy were summarized.
- The prospects in combination of chemotherapy and immunotherapy were discussed.
- The recent progress of nano-based drug delivery systems applied for cancer chemoimmunotherapy was further categorized and reviewed..

## Graphical abstract

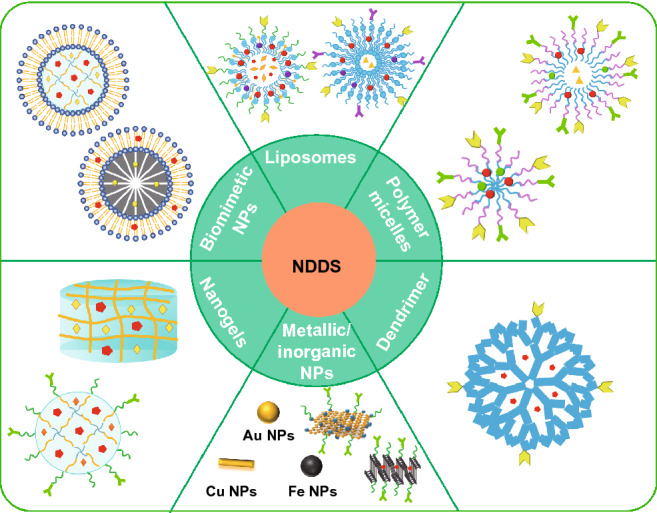

## Introduction

Cancer is still the main cause of death for patients worldwide with increasing incidence [1, 2], and the research into cancer treatment is under the spotlight. Surgery, radiotherapy, chemotherapy and immunotherapy, as well as those combinational regimens are now the main clinical strategies [3]. Among those, immunotherapy is now considered as the potentially powerful approach to overcome the cancer due to the completely different way for cancer treatment, which acts by modulating the systemic immune system rather than focusing on the tumor itself [4–7]. Since the first immune checkpoints blockade agent ipilimumab approved by the US Food and Drug Administration (FDA) in 2011, cancer immunotherapy has come of age and shown great clinical success [8]. Till now, additional six immune checkpoints blockade agents (Keytruda^®^, Opdivo^®^ Tecentriq^®^, Imfinzi^®^, Bavencio^®^ and Libtayo^®^) have been approved by FDA, and many other forceful immunotherapy drugs have been in clinical trials [9–12]. Nevertheless, immunotherapy has met great challenges in some tumor types or patients in clinical [13, 14], including drug resistance of immune checkpoints inhibitors, weak immunogenicity of therapeutic vaccines, significant immune-related adverse events (iRAE), off-target side effects [15] and so on.

Chemotherapy is the vital weapon of cancer therapy [16]. Chemotherapy drugs have long been considered to induce immune suppressive; however, massive preclinical studies proved that chemotherapy could offer additional benefits to immunotherapy, even some cytotoxic drugs could trigger antitumor immunity [17], such as cyclin-dependent kinases 4 and 6 inhibitor [18, 19]. Chemoimmunotherapy, the combination of chemotherapy and immunotherapy, provides a superior synergistic effect for enhancing antitumor efficiency. Firstly, chemotherapy drugs kill tumor cells directly, while immunotherapy reactivates immune response to kill cancer cells. Besides, the effective time was complementary, for which chemotherapy drugs have quick action but short action time, while immunotherapy could produce a strong and long-lasting antitumor effect. Additionally, immunotherapy could overcome the deficiencies on chemotherapy such as killing chemotherapeutical-resistant cells and cancer stem cells [13, 16]. Current data suggested that chemoimmunotherapy would bring incomparable prospects for optimizing the clinical prognosis of patients [20]. For example, the combination of carboplatin or cisplatin, pemetrexed with pembrolizumab, has been approved by FDA for the first-line treatment of non-small cell lung cancer (NSCLC).

To ensure optimal synergistic antitumor efficacy, some issues should be concerned, including distinct pharmacokinetics and in vivo distribution of both agents, insufficient tumor specificity and tumor accumulation, unascertainable drug ratios at tumor tissues and serious systemic side effects [21, 22]. Nano-based drug delivery system (NDDS) could improve the in vivo pharmacokinetics behaviors, increase the stability of drugs, realize the targeted delivery and controlled release of drugs, thus holding great promise for chemoimmunotherapy [23]. Moreover, recent studies demonstrated that nanoparticles (NPs) could re-model immunosuppressive tumor microenvironment (TME) [24]. Therefore, NDDS applied to chemoimmunotherapy is nowadays the hotspot in cancer treatment. Herein, the current approaches of cancer immunotherapy as well as chemoimmunotherapy were discussed. Next, the current applications of NDDS in chemoimmunotherapy were summarized.

## Cancer Immunotherapy

**Fig. 1:**
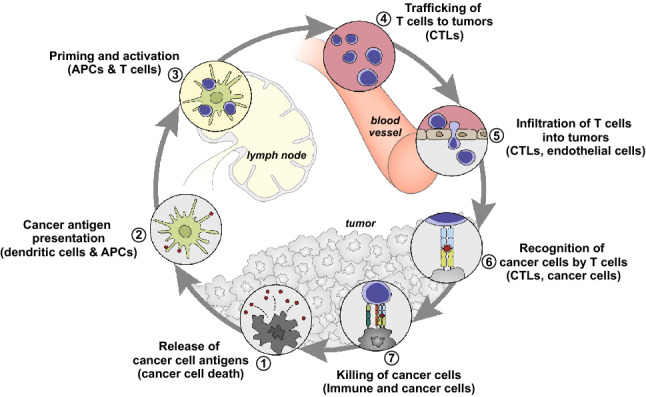
Scheme illustration of the cancer-immunity cycle. Reproduced with permission from Ref. [25]. Copyright 2013 Elsevier

Cancer immunotherapy has rapidly developed as a promising strategy for cancer treatment. Cancer immunity consists of several key steps, which is so-called cancer-immunity cycle, including release of cancer cell antigens, cancer antigens presentation by antigen-presenting cells (APCs), priming and activation of T cells, trafficking and infiltration of T cells to tumors and finally the recognition and killing of tumor cells by cytotoxic T cells (Fig. 1) [25]. It is theoretically possible that each step among them might be the potential therapeutic target with various methods. Aiming to these targets, current approaches to cancer immunotherapy mainly include therapeutic antibodies, cancer vaccines, adoptive cell therapy and cytokine therapy [26].

### Therapeutic Antibodies

At present, dozens of therapeutic antibodies, such as brentuximab vedotin (Adcetris^®^) and ibritumomab tiuxetan (Zevalin^®^) [27], have been approved by FDA for the treatment of various cancers, and some other therapeutic monoclonal antibodies (mAbs) were in clinical trials [28]. The therapeutic antibodies approved by FDA and the National Medical Products Administration (NMPA) for cancer immunotherapy are summarized in Table 1.

**Table 1:** Summary of therapeutic antibodies approved for cancer immunotherapy

Mechanism	Target	Drug	Type of cancer	Time to approved
Blockade of immune checkpoints	CTLA-4	Ipilimumab	Unresectable or metastatic melanoma	2011
	PD-1	Nivolumab	Classic Hodgkin’s lymphoma, Melanoma, non-small cell lung cancer and renal cell carcinoma	2014
	PD-1	Pembrolizumab	Advanced, unresectable or metastatic melanoma; non-small cell lung cancer and head and neck cancer	2014
	PD-1	Cemiplimab	Metastatic cutaneous squamous cell carcinoma or locally advanced cutaneous squamous cell carcinoma	2018
	PD-1	Toripalimab	Unresectable or metastatic melanoma	2018
	PD-1	Sintilimab	Classical Hodgkin’s lymphoma in patients who have relapsed or are refractory after ≥ 2 lines of systemic chemotherapy	2018
	PD-1	Camrelizumab	Relapsed or refractory classic Hodgkin’s lymphoma treated with at least second-line chemotherapy	2019
	PD-1	Tislelizumab	Relapsed or refractory classical Hodgkin’s lymphoma after at least second-line chemotherapy	2019
	PD-L1	Atezolizumab	Locally advanced or metastatic urothelial carcinoma	2016
	PD-L1	Durvalumab	Locally advanced or metastatic urothelial carcinoma	2017
	PD-L1	Avelumab	Metastatic Merkel cell carcinoma (mMCC)	2017

Notably, immune checkpoint therapy has brought significant clinical advances against cancer [29]. Immune checkpoints are vital for maintaining self-tolerance, regulating the duration and magnitude of immune response for immune system. By blocking immune checkpoints, immune checkpoint therapy can reactivate immune cells and enhance the killing ability of immune cells to cancer cells. Up to now, seven immune checkpoint agents have been approved by FDA, which are expected to be approved for various cancers in the future [30]. Ipilimumab (Yervoy^®^) is the first immune checkpoint agent, approved for the treatment of melanoma, which acts by blocking the cytotoxic T-lymphocyte-associated protein 4 (CTLA-4) and re-activating T cells. In stage IV melanoma patients, the mortality was reduced to 34% after treatment with ipilimumab plus dacarbazine, compared with dacarbazine plus placebo [31]. Besides, programmed death 1 (PD-1)/programmed death ligand 1 (PD-L1) antibody is another main category in immunotherapy, which could reactivate T cells by blocking the binding of PD-1 to PD-L1 and thus kill tumor cell indirectly. Nivolumab (Opdivo^®^), pembrolizumab (Keytruda^®^) and cemiplimab (Libtayo^®^) are PD-1 antibodies for the treatment of advanced melanoma [32], metastatic cutaneous squamous cell carcinoma (CSCC) [33], respectively. While on the other hand, atezolizumab (Tecentriq^®^), avelumab (Bavencio^®^) and durvalumab (Imfinzi^®^) are the PD-L1 blocking agents approved for the treatment of advanced or metastatic urothelial carcinoma, Merkel cell carcinoma (mMCC) [34] and urothelial carcinoma [35], respectively. Additionally, there are also other immune checkpoints as the potential targets in immune checkpoint therapy, such as T cell immunoglobulin and mucin-domain containing-3 (TIM-3), indoleamine-pyrrole 2,3-dioxygenase-1 (IDO-1) and lymphocyte-activation gene 3 (LAG-3), and their related antibodies are being evaluated in preclinical tumor models and/or in the clinic. At present, 4 immune checkpoint antibodies (toripalimab, sintilimab, camrelizumab and tislelizumab) developed in China have been approved by NMPA for cancer immunotherapy.

Recently, the combination of two immune checkpoints in tumor therapy showed a good prospect. For instance, the combination of PD-1 and CTLA-4 antibody has been used for melanoma immunotherapy. The response rate was 61%, and over 22% patients showing a complete response [36].

### Cancer Vaccines

The use of vaccines in prevention and treatment of cancers has been explored for more than a century. A remarkable progress has been achieved in the development of preventive vaccines—hepatitis B and human papilloma viruses (HPVs). HPVs vaccines have demonstrated definite potentials to prevent the cancers and saved millions of lives [37]. However, the development of therapeutic vaccines was painfully slow and faced numerous challenges. Nowadays, Sipuleucel-T (Provenge^®^) was the only therapeutic cancer vaccine approved by FDA, which is applied for the treatment of prostate cancer. Depending on the uptake of dendritic cells (DCs) and antigen presentation, cancer vaccines need to induce antigen-specific CD8^+^ cytolytic T cells (CTLs) and antigen-specific CD4^+^ T cells for optimally efficacy [38].

With the deep understanding of tumor immunology and the success of Sipuleucel-T, several types of cancer vaccines and other diverse vaccines have now been evaluated in phase II and phase III clinical trials [39, 40], such as granulocyte–macrophage colony-stimulating factor (GM-CSF) gene-modified autologous tumor vaccine (CG8123), peptide-based glycoprotein 100 (Gp100), TGF-β2 antisense/GM-CSF gene-modified autologous tumor cell vaccine (TAG) and New York esophageal carcinoma antigen 1 Plasmid DNA (pPJV7611) [41–43]. Cancer vaccines often combined with adjuvants to produce powerful immune responses [44]. The commonly used adjuvants mainly include cytokines/endogenous immunomodulators (e.g., GM-CSF), microbes and microbial derivatives (e.g., cytosine-phosphate diesterguaninen (CpG), poly I:C), mineral salts (e.g., alum), viral vectors (e.g., adenovirus, vaccinia), oil emulsions or surfactants (e.g., Montanide™) and so on [38, 45–48].

### Adoptive Cell Therapy

Adoptive cell therapy (ACT) relies on the ex vivo generation of highly active and tumor-specific lymphocytes, and then a large number of these lymphocytes were injected to the autologous hosts for cancer treatment [49]. These lymphocytes mainly include lymphokine-activated killer cells (LAK cells), chimeric antigen receptor T cells (CAR-T cells), tumor-infiltrating lymphocytes (TIL), natural killer (NK), DCs, macrophages and so on.

ACT has multiple advantages compared with other cancer immunotherapeutic approaches. Large numbers of antitumor lymphocytes can be readily grown in vitro and recognize the tumor specifically, then play effective antitumor immune effect [50]. For example, anti-CD19 CAR-T cells showed high antitumor efficacy in patients with relapsed B-cell acute lymphoblastic leukemia (B-ALL) and B-cell non-Hodgkin lymphoma, and the complete response rate was 70–94% in various trials [51]. Two kinds of ACT with anti-CD19-modified T cells have been approved by FDA, i.e., Kymriah™ and Yescarta^®^ [52], which marked an era arriving of ACT. Suitable NDDS have been applied for adoptive cell therapy. For example, Mitragotr et al. [53] developed an engineered particle (name as “backpack”), which could robustly bind on the surfaces of macrophages and regulate the phenotypes of macrophages by sustained releasing cytokines in vivo. The backpack-loaded macrophages could keep antitumor phenotypes for up to 5 days and showed superior antitumor effect compared with free cytokine-treated macrophages.

### Cytokines

Cytokines with biological activity could enhance the immune response of patients via inducing direct apoptosis effects and generate the antitumor effects indirectly [54, 55]. Different cytokines have been intensively applied in clinical cancer treatment, such as interferons (INFs), interleukins (ILs), tumor necrosis factors (TNFs) and granulocyte-macrophage colony-stimulating factor (GM-CSF) [56].

IFN-α was the first cytokine approved into market in 1995 and used for leukemia and advanced melanoma. IFN-γ has properties of immunomodulation, which could induce the expression of MHC-I/II by APC, activate NK/macrophages and induce the differentiation of T cells [55, 57]. IL-2, a multifunctional cytokine, is essential in differentiation and proliferation of T cells, NK, macrophages and B cells, which is effective in metastatic melanoma and renal carcinoma [58]. Other lymphokines are also being evaluated, such as IL-7, IL-12 and IL-15 [57]. TNFs are one kind of most active biological factors found to kill tumor directly [59, 60]. GM-CSF showed potential to promote the growth and differentiation of macrophages/granulocytes/DCs, which could enhance antigen presentation [61].

Although revealing obvious advantages, immunotherapy has met great challenges in some tumor types or patients in clinics, including drug resistance of immune checkpoints inhibitors, weak immunogenicity of therapeutic vaccines, significant immune-related adverse events (iRAE) and off-target side effects etc. [62].

## Cancer Chemoimmunotherapy

### Cancer Chemotherapy

Caner chemoimmunotherapy is a promising approach for improving antitumor efficiency and has been widely studied in preclinical and clinical research. Chemotherapy is one of the most used cancer treatments, which offers the best hope of cancer. Chemotherapy takes effect by toxic compounds that inhibit the fast proliferation of cancer cells [63]. Unfortunately, other rapid growth cells may be inhibited by chemotherapeutic drugs as well, such as hair follicles cells, bone marrow cells and gastrointestinal tract cells. Thus, toxic side effects of chemotherapy usually include hair loss, severe nausea and bowel problems, etc. Chemotherapy frequently fails in cancer treatments due to poor pharmacokinetics and wide distribution in vivo, insufficient delivery and multiple drug resistance (MDR) [64]. At present, combination therapy was used to enhance the curative effect of chemotherapy, such as chemotherapy combined chemotherapy, surgical treatment, radiotherapy, photothermal therapy [65], photodynamic therapy [66], immunotherapy [67] and so on. Among these, the combination of chemotherapy and immunotherapy (chemoimmunotherapy) provides a superior synergistic effect for enhancing antitumor efficiency.

### How Chemotherapy Influence Cancer Immunotherapy?

Chemotherapy drug might induce immunomodulation mainly by enhancing intrinsic tumor cell immunogenicity [68], regulating the suppressive influence of T cells [69] and impacting the function of other cells, such as myeloid-derived suppressor cells (MDSCs) [70] and DCs [71]. Studies have shown that chemotherapeutics could enhance intrinsic immunogenicity of tumor cells by upregulating the expression of tumor antigens [72] and MHC-I [73], inducing the expression of costimulatory molecules [74], downregulating the immune checkpoint molecules expressed on the tumor cell surface [75], inducing tumor cell death by secreting ATP or expressing calreticulin [68] and so on [76, 77].

Chemotherapeutic agents in appropriate doses can regulate the suppressive influence of tumor-associated T cells. Regulatory T cells (Tregs) are immunosuppressive CD4+ T cells and usually downregulate the proliferation of effector T cells. The numbers of Tregs account for only about 4% of total CD4+ T cells, while up to 20–30% of total CD4+ T cells in TME, that would suppress the antitumor immune remarkably [78–80]. Studies have demonstrated that some chemotherapeutic agents can regulate the suppression of Tregs to a certain extent [81]. For example, selective CDK4/6 inhibitors (such as abemaciclib) could promote antitumor immunity by two aspects [19]. On the one hand, CDK4/6 inhibitors stimulate the production of IFN-III and enhance tumor antigen presentation. On the other hand, CDK4/6 inhibitors could suppress the proliferation of Tregs. In a study by combining abemaciclib with PD-L1 inhibitor for the treatment of MMTV-rtTA/tetO-HER2 tumors in mice, the tumor volumes reduced ~ 70% by day 13.

With the advantages of chemoimmunotherapy, numerous clinical trials have shown delightful results in cancer treatment (Table 2). Chemoimmunotherapy exhibited remarkable clinical outcomes of cancer patients. For example, E. Ellebaek et al. analyzed metastatic melanoma patient’s treatment with DC vaccination plus cyclophosphamide/celecoxib/IL-2. Compared with treatment without cyclophosphamide and celecoxib, the 6-month survival increased significantly [82]. Nowak et al. had explored the immunological effect of CD40-activating antibody with cisplatin/pemetrexed in malignant pleural mesothelioma, in which more patients showed transient tumor-specific T cell responses and achieved long-term survival [83]. The combination of carboplatin or cisplatin, pemetrexed/pembrolizumab, was approved by FDA for the first-line treatment of NSCLC.

**Table 2:** Summary of clinical trials for cancer chemoimmunotherapy

Clinical trial	Immunotherapy drug	Chemotherapy drug	Type of cancer	References
Phase 1b	CD40-activating antibody CP-870,893	Cisplatin and pemetrexed	Malignant pleural mesothelioma	[83]
Phase 2	Ipilimumab	Paclitaxel and carboplatin	Non-small cell lung cancer	[76]
Phase 2	Cox-2 inhibitor, granulocyte colony-stimulating factor, a sulfhydryl (SH) donor and a hemoderivative	Cyclophosphamide	Pancreatic adenocarcinomas, non-small cell lung cancer or prostate cancer	[161]
Phase 2	Bevacizumab	Cyclophosphamide	Advanced ovarian cancer patients	[84]
Phase 2	Oncolytic adenovirus	Cyclophosphamide	Advanced soild tumors	[81]
Phase 2	Oncolytic adenovirus	Temozolomide	Advanced soild tumors	[162]
Phase 2	Interleukin-2	Cyclophosphamide	Metastatic melanoma	[82]
Phase 3	GM-CSF + telomerase peptide vaccine GV1001	Gemcitabine/capecitabine	Advanced pancreatic cancer	[87]

As shown in recent clinical trials (Table 2), the addition of chemotherapeutic drugs to immunotherapy could synergistically increase the antitumor effects compared with either therapy alone [84, 85]. Nevertheless, some clinical trials of antitumor effect are still not ideal, such as inadequate T cells response, great differentiation in curative effect among tumor patients, and many patients do not have a good response [86]. Middleton et al. conducted a phase 3 clinic trial of telomerase peptide vaccine (GV1001) plus chemotherapy drug in pancreatic cancer to assess the efficacy and safety. Results interpreted that GV1001 vaccine plus chemotherapy didn’t improve overall survival [87]. It was suggested that new approaches to further enhance the immune response effects during chemoimmunotherapy are explored.

## Nanocarriers for Cancer Chemoimmunotherapy

NDDS provides promising strategies for cancer chemoimmunotherapy, because they are easy to be internalized by immune cells and could re-educate TME due to special physical and chemical properties, thus boost the immune system [88]. NDDS can increase solubility and bioavailability of the agents, prolong the circulation time of agents via passive or active targeting, increase the accumulation of therapeutic agents in tumor tissue as well as improve the pharmacokinetics behaviors in vivo, leading to enhanced therapeutic effects and reduced side effects [89–92].

Concerning NDDS applied to chemoimmunotherapy, there are several flexible approaches to realize the co-delivery of multiple agents [93, 94]. For the combination of multiple agents in chemoimmunotherapy, one agent can be administered as free form and others by NDDS (Free drug + Nano), or both were delivered by similar or different NDDS, respectively (Nano + Nano), or both agents were co-encapsulated in one NDDS (co-encapsulation). The advantages and disadvantages of the three approaches to deliver multiple agents in chemoimmunotherapy are summarized in Table 3.

**Table 3:** Advantages and disadvantages of the three approaches in chemoimmunotherapy

Approach	Advantages	Disadvantages
“Free drug + nano” approach	1. Adjustable prescription 2. Adjustable administration interval 3. Easy for preparation 4. Easy for industrial scale-up	1. Undesired distribution of two agents in vivo 2. Uncontrolled onset time 3. Insufficient tumor selectivity 4. Potential systemic toxicity
“Nano + nano” approach	1. Flexibility in formulation 2. Adjustable administration dosing 3. Coordinated distribution of two agents in vivo	1. Mismatched half-lives and in vivo pharmacokinetics 2. Uncontrollable onset time in tumor tissue
Co-encapsulation approach	1. Uniform distribution of the drugs in vivo 2. Accumulation in the tumor tissue at proper ratio 3. Drug release in a controlled manner 4. Controlled temporal and spatial delivery of multiple agents	1. Complex preparation process 2. Difficulty in delivering to different targets

The “Free drug + Nano” approach is the closest to the current treatment of cancer with nanomedicines. The “Free drug + Nano” approach exhibited advantages of adjustable prescription, controllable administration interval, easy for preparation, easy industrial scale-up and clinical transformation [95], which mainly include two strategies. One is that immunotherapeutic agent can be delivered in appropriate NPs, and the chemotherapy drug was administered in free form. Yong Taik Lim et al. have designed two poly(lactic-co-glycolic acid) (PLGA) NPs combined with chemotherapy drug paclitaxel (PTX), one is CpG-loaded PLGA NPs (PCNs) to activate BMDCs, the other is IL-10 small interfering RNA-loaded PLGA NPs (PINs) to silence IL-10 [96]. The treatment of PTX followed by PCNs and PINs could enhance antitumor effect and increase survival rate in B16-F10-bearing melanoma mice compare to PTX alone (*p *< 0.05). Another “Free drug + Nano” approach was that immunotherapy agent was administrated in free form, and the chemotherapy drug was loaded in NDDS [97]. Li et al. have reported TME-activatable prodrug vesicle for co-loading oxaliplatin (OXA) prodrug and photosensitizer (PS), which would produce immunogenic cell death (ICD) of the tumor cells [98]. The prodrug vesicle was combined with αCD47-mediated CD47 blockade for antitumor immunity of ICD. The results showed that prodrug vesicle-mediated ICD and CD47 blockade could inhibit tumor growth, suppress metastasis and recurrence of tumor.

The “Nano + Nano” approach might be flexible in formulation, adjustable in administration dosage and have coordinated distribution of two agents [99, 100]. Our group had developed two twin-like NPs (TCN) for different cells targeting delivery of sorafenib (SF) and IMD-0354 to enhance tumor-localized chemoimmunotherapy [99]. The two TCN exhibited coordinated distribution in vivo and could realize the targeting delivery into different cells, thus ensuring superior synergistic antitumor efficacy and M2-type macrophages polarization ability. Lin et al. developed two-type CD44-targeted liposomes, one for anti-IL6R antibody encapsulating for immunotherapy and the other for DOX encapsulating for chemotherapy to inhibit the metastasis of breast cancer [101]. The NPs-αIL6R Ab-CD44 specifically modified the immune environment in primary tumor by inhibiting the infiltration of TAMs to form a tumor microenvironment unfavorable for metastasis and achieved a significant effect to inhibit the metastasis of breast cancer. For another example, Li et al. designed low molecular weight heparin (LMWH)-D-α-tocopherylsuccinate (TOS) micelles (LT) encapsulating chemotherapeutic drug DOX (LT-DOX) or Toll-like receptor 7 agonist imiquimod (LT-IMQ) with PD-L1 immune checkpoint blockade for chemoimmunotherapy for the treatment of metastatic breast cancer [102]. The two micelles could prolong the circulation time and increase the accumulation in tumor. LT-DOX could initiate a tumor-specific immune response by eliciting ICD, which further strengthened by adjuvant LT-IMQ. The combination with clinically approved PD-L1 checkpoint blockade inhibited the activities of Treg cells, which alleviated the immune inhibition signal and promoted antitumor efficacy.

**Fig. 2:**
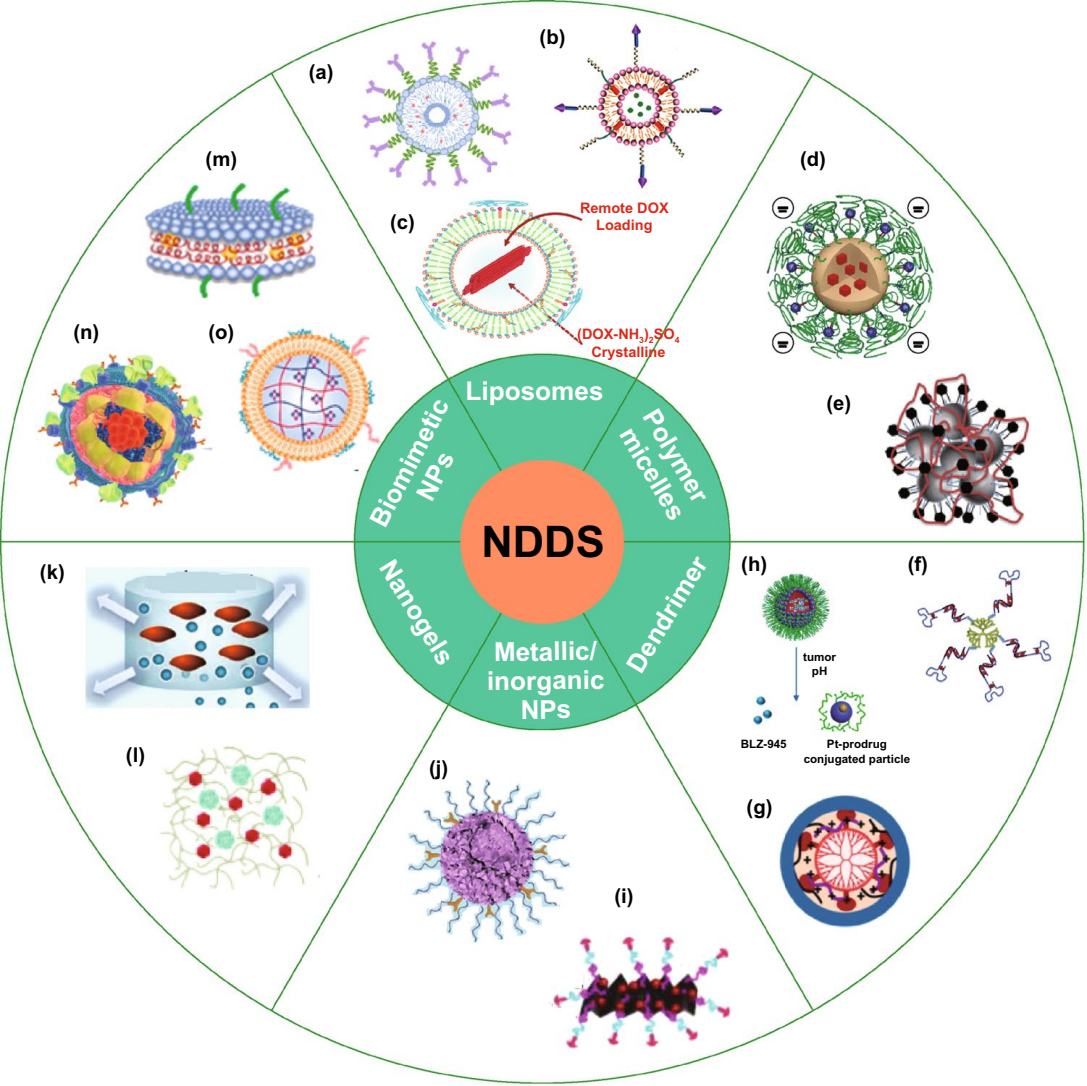
Summary of NDDS for “co-encapsulation” approach in chemoimmunotherapy. Liposomes, reproduced with permission from Ref. **a** [104], Copyright 2018 Springer; **b** [105], Copyright 2019 American Chemical Society; **c** [106], Copyright 2018 American Chemical Society. Polymer micelles, reproduced with permission from ref **d** [107], Copyright 2020 John Wiley and Sons; **e** [108], Copyright 2015 John Wiley and Sons. Dendrimer, reproduced with permission from Ref. **f** [109], Copyright 2011 Elsevier; **g** [110], Copyright 2019 lvyspring International Publisher; **h** [111], Copyright 2017 American Chemical Society. Inorganic NPs and Metallic NPS, reproduced with permission from Ref. **i** [112], Copyright 2017 American Chemical Society; **j** [113], Copyright 2019 John Wiley and Sons. Nanogels, reproduced with permission from Ref. **k** [114], Copyright 2017 Elsevier; **l** [115], Copyright 2020 Elsevier. Biomimetic NPs, reproduced with permission from Ref. **m** [116], Copyright 2019 American Chemical Society; **n** [117], Copyright 2019 American Chemical Society; **o** [118], Copyright 2017 American Chemical Society

The “Free drug + Nano” and “Nano + Nano” approaches still suffered from potential mismatched in vivo pharmacokinetics and uncontrolled onset time in tumor tissue. The “co-encapsulation” approach could uniform the distribution of drugs in vivo, control the accumulation in tumor tissue at proper ratio, ensure the drug release in a controlled manner and controlled temporal and spatial delivery of multiple agents [103]. Abundant NDDS have been designed for “co-encapsulation” approach in chemoimmunotherapy, including liposomes, polymer micelles, dendrimers, metallic and inorganic NPs, nanogel and biomimetic NPs (Fig. 2). Moreover, the representative NDDS application to chemoimmunotherapy is summarized in Table 4.

**Table 4:** Summary of NDDS for “co-encapsulation” approach in chemoimmunotherapy

Type of carrier	Chemotherapy drug	Immunotherapy	In vitro cell line or in vivo tumor model	References
Liposomes	Doxorubicin	PD-L1 inhibitor	B16F10 tumor-bearing C57BL/6 mice	[105]
Liposomes	Doxorubicin	Indoximod	4T1 cells orthotopic breast cancer models	[106]
Liposomes	Docetaxel	PD-L1 mAb	B16-F10 cells xenograft tumor animal model	[104]
Polymer micelles	All-trans retinoic acid	PD-L1 mAb	C3H tumor-bearing mice	[128]
Polymer micelles	Dacarbazine	DR5 mAb	A375 cells and NIH cells	[163]
Polymer micelles	Dacarbazine	DR5 mAb	A375 BALB/c nude mouse tumor model	[164]
Polymer micelles	Paclitaxel	HY19991	MCF-7 tumor-bearing mice	[137]
Polymer micelles	Curcumin	NLG919	B16F10 tumor-bearing C57BL/6 mice	[129]
Polymer micelles	Paclitaxel	PD-L1 mAb	B16F10 tumor-bearing C57BL/6 mice	[107]
Polymer micelles	Pt(IV) prodrug	poly(I:C)	PC3, MDA-MB231, PANC-1 cells	[108]
Dendrimers	Doxorubicin	CpG	22RV1 cells BALB/mice	[109]
Dendrimers	Doxorubicin	CpG	B16-F10 melanoma-bearing mice	[110]
Dendrimers	Platinum	BLZ-945	CT26 colon cancer, B16 melanoma models and 4T1 tumor-bearing mice	[111]
Black phosphorus NPs	Doxorubicin	PD-L1 mAb/small interfering RNA	MC-38 tumor-bearing mice	[112]
CuS NPs	Docetaxel	CpG	Negative breast cancers	[113]
Hydrogel	Celecoxib	Anti-PD-1 mAb	B16-F10 melanoma and 4T1 metastatic breast cancer	[149]
Hydrogel	Cisplatin	IL-15	B16-F10 melanoma-bearing mice	[114]
Nanogel	Docetaxel	NLG919	4T1-Luc murine breast cancer xenograft mouse model	[150]
Hydrogel	Doxorubicin	CpG	B16 melanoma-bearing mice	[115]
Hydrogel	Doxorubicin	IL-2/IFN-g	B16-F10 melanoma-bearing mice	[151]
Hydrogel	Doxorubicin	melittin-RADA32	B16-F10 melanoma-bearing mice	[152]
Albumin biomimetic NPs	Temozolomide	Regorafenib	U87 orthotopic glioma-bearing mice	[153]
HLD-biomimetic nanodiscs	Docetaxel	CpG	Glioblastoma	[116]
Lactoferrin NPs	Shikonin	JQ1	CT26 tumor-bearing mice	[154]
Erythrocyte membrane biomimetic NPs	Paclitaxel	IL-2	B16–F10 melanoma-bearing mice	[118]
Tumor cell membrane biomimetic NPs	Doxorubicin	Surface-layer protein (adjuvant)	B16–F10 melanoma-bearing mice	[117]
NK cell membrane biomimetic NPs	Oxaliplatin	1-Methyl-d-tryptophan	4T1 tumor-bearing mice	[160]

### Liposomes

Liposomes are the bilayer vesicles composed of phospholipids and cholesterol, which possess advantage of high encapsulation efficiency, targeting ability and low toxicity, holding great prospects in industrial production. The immunotherapy agents, like antigens and adjuvants, can be encapsulated in the hydrophobic core or adsorbed on the lipid surface via charge interactions between agents and lipid or with a chemical linker to the lipid bilayer [119]. Meanwhile, hydrophilic small-molecule chemotherapeutic agents can be encapsulated in the interior aqueous cores, in which hydrophobic agents can be encapsulated into lipid bilayers [104].

**Fig. 3:**
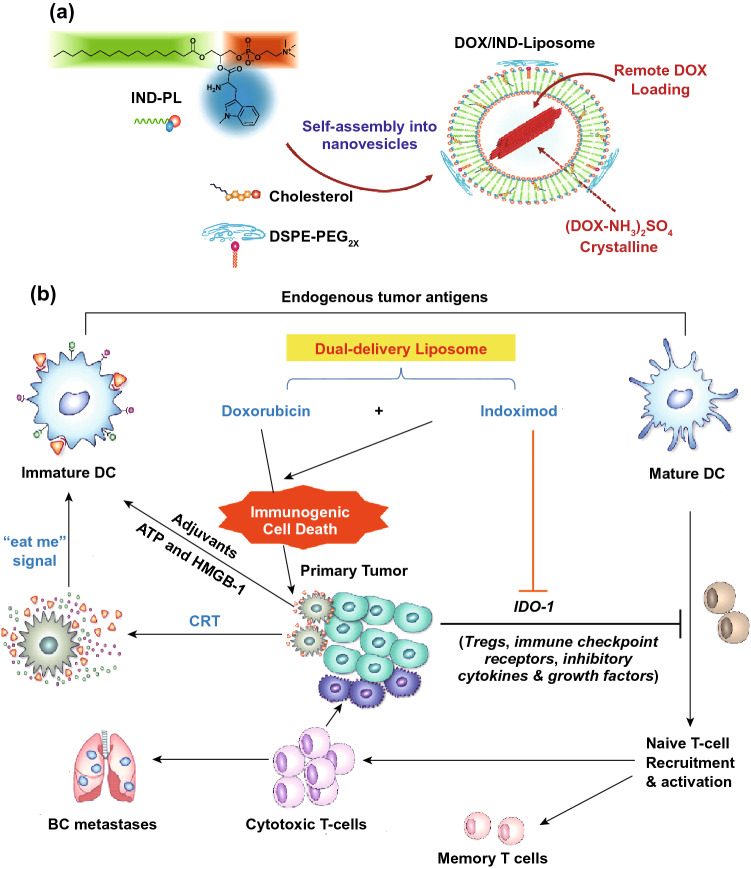
**a** Synthesis of DOX/IND-liposome, **b** Illustration of breast cancer immunotherapy by combined delivery of DOX plus IND. Reproduced with permission from Ref. [106]. Copyright 2018 American Chemical Society

Liposomes have been well applied in cancer therapy with kinds of liposomal products approved. Meanwhile, liposomes were also widely studied as the vehicles to exert the maximal efficacy of chemoimmunotherapy. Chen et al. developed pH and matrix metalloproteinases (MMPs) dual responsive liposomes (LPDp) with PD-L1 inhibitor conjugate combined with low-dose chemotherapy doxorubicin (DOX) to achieve enhanced antitumor efficacy [105]. LPDp achieved the optimal tumor suppression efficiency (∼ 78.7%) due to synergistic contribution of chemotherapeutic agents and the blockade of immune checkpoints. Lu et al. [106] have established a liposome for co-loading DOX and IDO-1 inhibitor indoximod (IND) for chemoimmunotherapy (DOX/IND-liposome). DOX/IND-liposome was self-assembled by phospholipid-conjugated IND, followed by the remote DOX loading (Fig. 3). The results demonstrated that DOX/IND-liposomes enhanced the anti-breast cancer immune response significantly than DOX-liposomes.

### Polymer Micelles

Polymer micelles are thermodynamically stable colloidal solutions formed by self-assembly of amphiphilic block copolymers [120]. Hydrophobic small-molecule drugs could be encapsulated in the hydrophobic core of micelles, and hydrophilic drugs could be loaded via physical interactions or chemical conjugation [121]. Genexol^®^-loaded PTX and Nanoxel^®^-loaded docetaxel (DTX) have been approved for the cancer treatment. Polymer micelles have been widely evaluated in cancer chemoimmunotherapy. Furthermore, the multifunctional polymer micelles can be obtained by modification on the surface of polymeric materials, which could package hydrophilic or hydrophobic drugs efficiently and protect them from degradation in vitro and in vivo.

PLGA and polylactic acid (PLA) are FDA approved polymers materials with biodegradable and biocompatible features [122–124]. Polymer micelles prepared from PLGA/PLA have been evaluated as drug carriers in chemoimmunotherapy [125–127]. For example, Zhou et al. [128] have developed a PLGA-PEG micelle co-delivering all-trans retinoic acid (ATRA) and PD-L1 mAb for the treatment of oral dysplasia and oral squamous cell carcinoma. Antitumor assay in vivo demonstrated that the ATRA-PLGA-PEG-PD-L1 had superior therapeutic efficacy than free ATRA and CD8^+^ T cells were activated in TME after treatment.

**Fig. 4:**
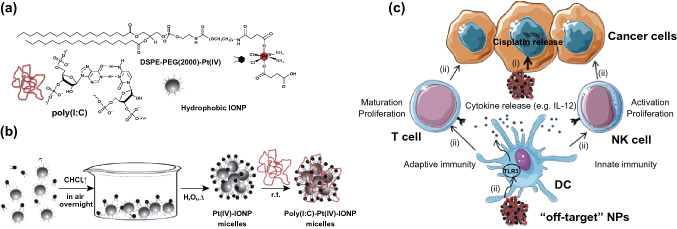
Preparation of the poly (I:C)–Pt(IV)–IONP micelles. **a** Structure and schematic representation of the reaction components and **b** schematic of the simple self-assembly procedure used to prepare the Pt(IV)–IONP micelles and poly (I:C)–Pt(IV)–IONP micelles (not to scale). **c** Schematic illustration of the combination therapeutic effect of poly (I:C)-Pt(IV)-IONPs. Reproduced with permission from Ref. [108]. Copyright 2015 John Wiley and Sons

Other multifunctional polymer micelles were also explored to improve the efficacy of chemoimmunotherapy. Xintao Shuai and his group designed pH and MMP-2 dual-sensitive micelles to co-deliver anti-PD-1 antibody (aPD-1) and PTX for synergistic cancer chemoimmunotherapy [107]. The micelles showed an enhanced tumor chemoimmunotherapy effect in murine melanoma model. Juan C. Mareque-Rivas and co-workers reported Pt(IV) prodrug-modified PEGylated phospholipid micelles that encapsulate iron oxide NPs (IONPs), which were functionalized with poly (I:C) for chemoimmunotherapy (poly (I:C)-Pt(IV)-IONPs) (Fig. 4) [108]. The poly (I:C)-Pt(IV)-IONPs enhanced cytotoxicity in different tumor cells significantly and activated DC by cisplatin and poly (I:C) in immunotherapy greatly. In a study by Yang et al., size-shrinkage and charge-reversal micelles co-loaded IDO inhibitor NLG919 and curcumin (CUR) were developed (PCPCD) [129]. PCPCD showed high efficiency of inhibiting tumor growth, metastasis and recurrence in vivo by the combined effects of chemotherapy-enhanced immunogenicity, and NLG919-induced IDO-blockade immunotherapy.

### Dendrimers

Dendrimers are hyperbranched spherical polymers formed of a hydrophobic central core, branched monomer and functional peripheral groups [130]. With the unique structural features of dendrimers, such as structural clarity, close-to-monodisperses, ease of multi functionalities and multivalences, numerous novel dendrimer-based NPs have been designed and attracted scientific attention [131–133]. The hydrophobic central core could be loaded with small molecular drugs and the functional peripheral group can chemically link immunotherapy agents, such as therapeutic antibody. At present, the most widely used dendrimers are polyamidoamine (PAMAM), polypropyleneimine (PEI) and peptide dendrimers.

**Fig. 5:**
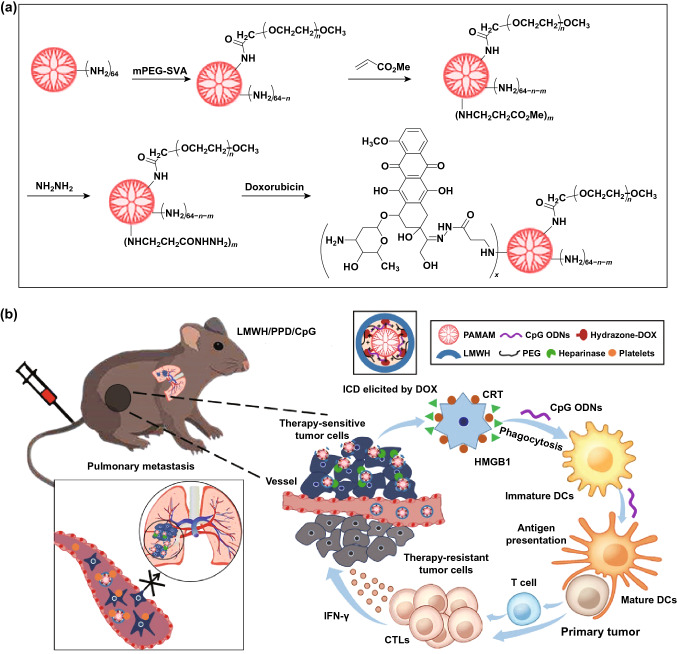
**a** Schematic illustration of reaction scheme for the synthesis of pH-sensitive hydrazone bond linked PEG-PAMAM-DOX conjugates (PPD). **b** Schematic illustration of LMWH/PPD/CpG to inhibit melanoma tumor. Reproduced with permission from Ref. [110]. Copyright 2019 lvyspring International Publisher

At present, several dendrimers have reached clinical trials for cancer immunotherapy, and they also have high application prospect in chemoimmunotherapy [109, 134–136]. He et al. [110] designed a PAMAM-based chemoimmunotherapy NPs (LMWH/PPD/CpG) by co-loading DOX and CpG for the treatment of metastatic melanoma. DOX conjugated on the amino-terminated PAMAM dendrimer by pH-sensitive hydrazone bond (PPD). LMWH/PPD/CpG were formed by negatively charged low molecular weight heparin (LMWH) coating on the surface of PAMAM. LMWH/PPD/CpG showed enhanced immune response in vivo and increased antitumor efficacy against melanoma (Fig. 5).

**Fig. 6:**
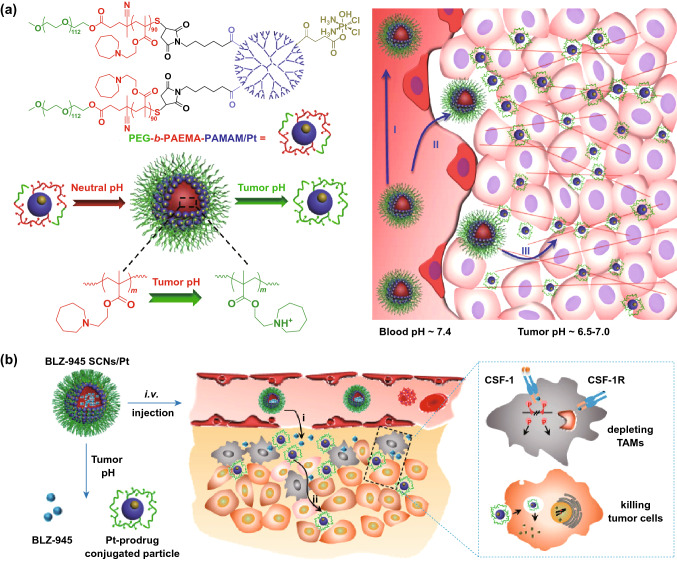
**a** Schematic illustration of PEG-b-PAEMA-PAMAM/Pt, reproduced with permission from Ref. [138]. Copyright 2016 American Chemical Society. **b** Mechanism of spatial delivery of BLZ-945 and Pt-prodrug to TAMs and tumor cells. Reproduced with permission from Ref. [111]. Copyright 2017 American Chemical Society

Currently, new dendrimers-based NPs have been used to achieve the deep penetration of loaded drugs for efficient chemoimmunotherapy [137]. Wang et al. reported a pH-sensitive poly(ethylene glycol)-*b*-poly(2-azepane ethyl methacrylate) amphiphilic block copolymer (PEG-*b*-PAEMA), which was further modified with PAMAM/Pt to obtain PEG-*b*-PAEMA-PAMAM/Pt NPs (SCNs/Pt) (Fig. 6a) [138]. The SCNs/Pt could achieve ultrasensitive size switching in the acidic TME for improved tumor penetration in vivo. Then, the SCNs was used for loading BLZ-945 (small-molecule inhibitor of CSF-1R of TAMs) and Pt-based prodrug NPs ^BLZ‑945^SCNs/Pt (Fig. 6b) [111]. Antitumor study in vivo showed that ^BLZ‑945^SCNs/Pt could inhibit the tumor growth more effectively, compared with ^BLZ‑945^SCNs or SCNs/Pt monotherapy.

### Metallic and Inorganic NPs

Metallic and inorganic NPs have shown favorable advantages as drug carriers, such as high drug loading capacity, feasibility of functionalization and no immunogenicity. A large number of metallic and inorganic NPs have been investigated for chemoimmunotherapy, including graphene oxide-based NPs (GO NPs) [139], mesoporous silica NPs (MSN NPs) [140–143], black phosphorus (BP) NPs [112], gold NPs (AuNPs), copper-derived NPs (Cu NPs) and so on [144, 145].

Taking BP as an example, BP is a new member of two-dimensional materials, nonmetallic-layered semiconductor with corrugated crystalline and textural properties. The unique structure enables BP has special properties, such as huge surface area, good mechanical flexibility, ultra-high photothermal conversion efficiency, good biocompatibility and biodegradability. BP showed a good prospect in photoacoustic imaging, photothermal therapy [146], photodynamic therapy and drug loading for chemoimmunotherapy [145, 147]. For example, Jong Oh Kim and co-workers reported coarse BP flakes with plug-and-play and ultrasonic bubble bursting features to load DOX, programmed death ligand 1 and small interfering RNA (BP-DcF@sPL) for chemo-photoimmunotherapy of colorectal cancer [112]. BP-DcF@sPL showed significantly prolonged and lasted survival period in MC-38 tumor xenografted in C57BL/6 mouse models.

**Fig. 7:**
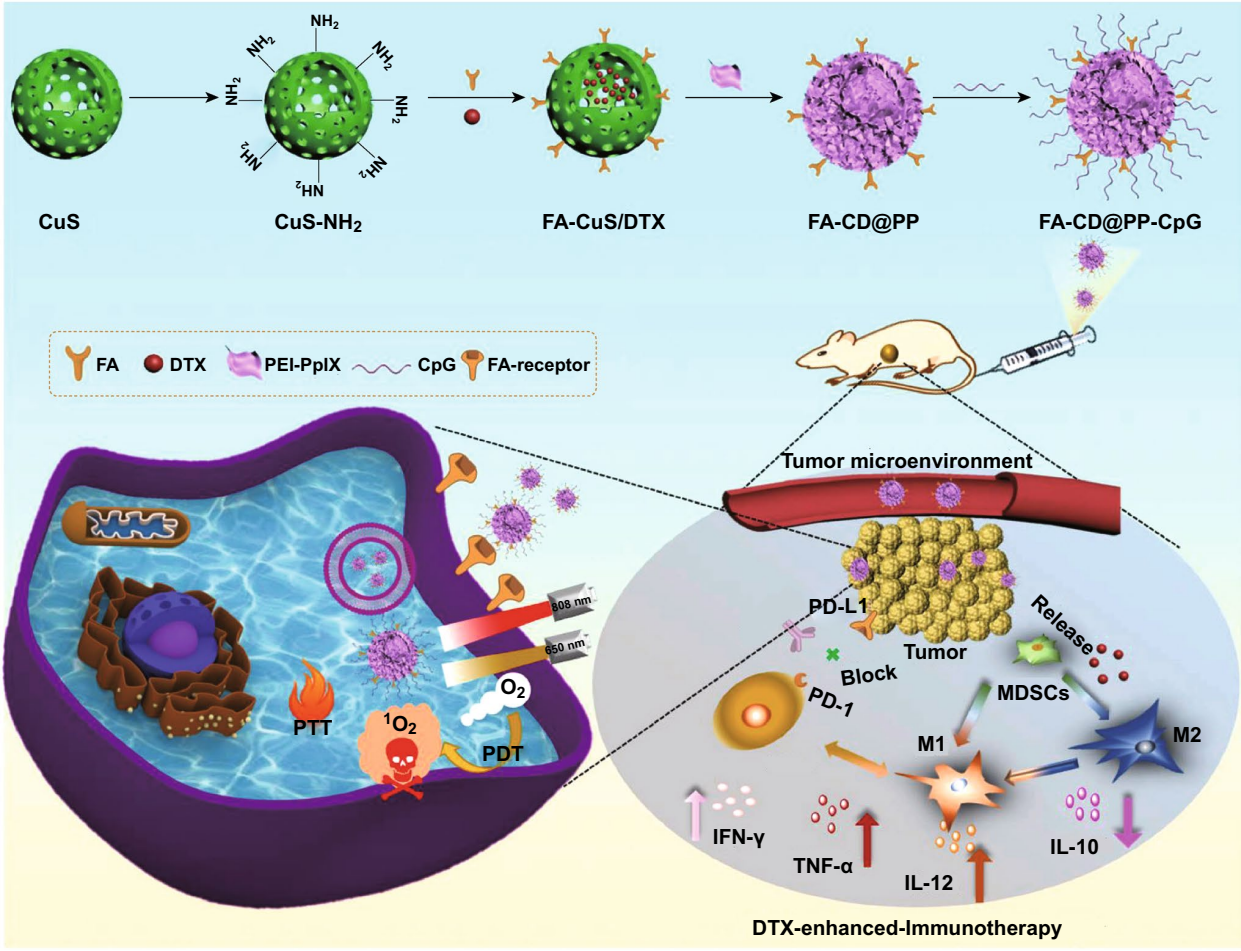
Scheme of the rational design and synthesis of FA-CD@PP-CpG and illustration of FA-CD@PP-CpG for DTX-enhanced immunotherapy. Reproduced with permission from Ref. [113]. Copyright 2019 John Wiley and Sons

Metallic material-derived NPs usually have photothermal therapy (PTT) and photodynamic therapy (PDT) effects, which not only can be used as photosensitizer, but also have great potential for cancer immunotherapy due to ICD. For example, Chunyan Dong and his group designed a multifunctional NPs FA-CuS/DTX@PEI-CpG NPs (FA-CD@PP-CpG) for synergistic PDT, PTT and DTX-enhanced immunotherapy [113]. FA-CD@PP-CpG can improve immunotherapy effect, such as promote infiltration of CTLs, suppress MDSCs and enhance antitumor efficacy on 4T1-tumor-bearing mice (Fig. 7).

### Nanogels

**Fig. 8:**
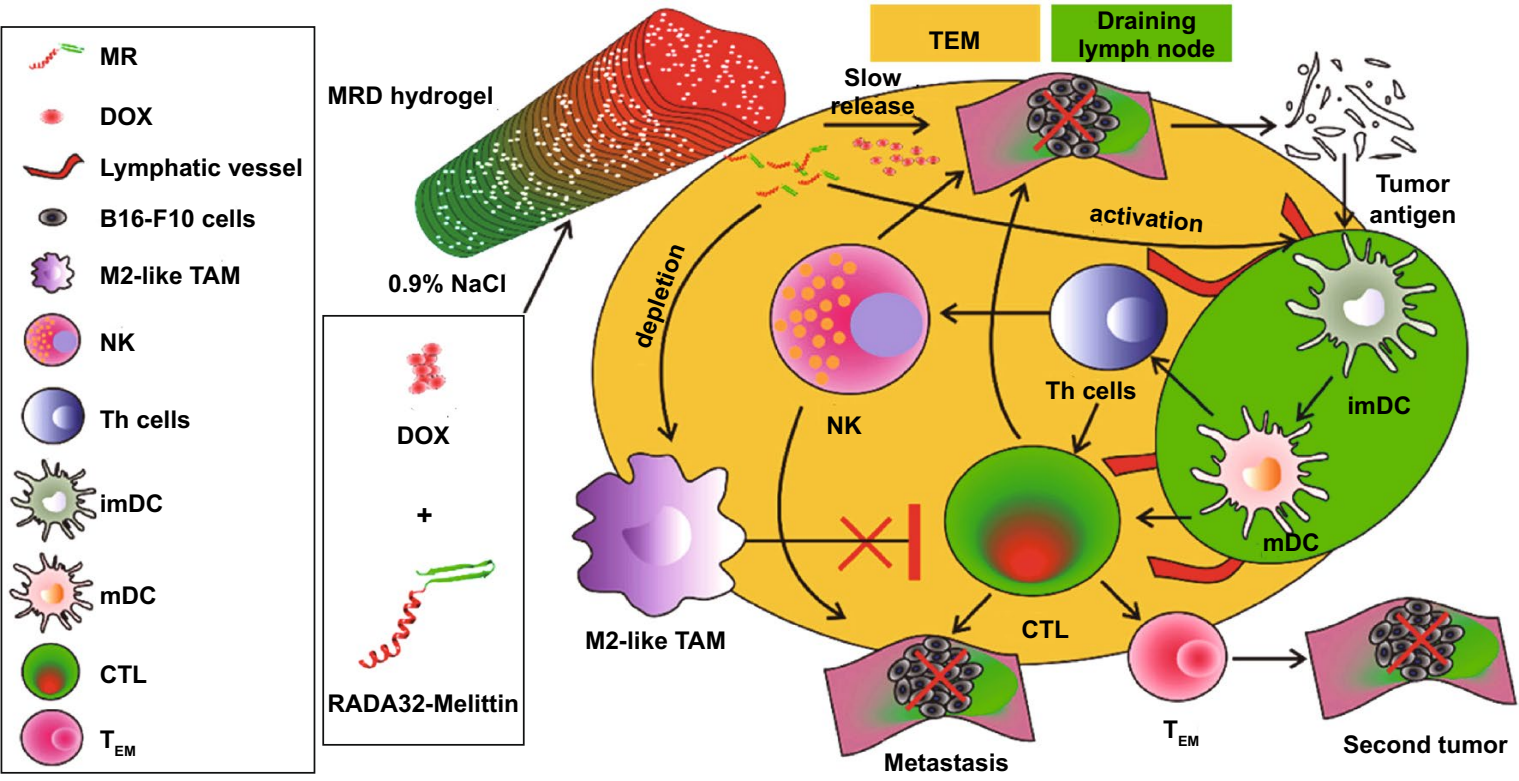
Mechanism of MRD hydrogel-mediated antitumor effects against melanoma. Reproduced with permission from Ref. [152]. Copyright 2018 American Chemical Society

Nanogels, with nano-sized hydrogel scaffold, good biocompatibility, high water contents and great compatibility with various therapeutic agents (such as small-molecule drugs and bio-macromolecules) have been considered as promising NDDS for effective chemoimmunotherapy. Multifunctional nanogels could be rationally designed for chemoimmunotherapy, by decorating with targeting ligands, synthesizing responsive functional bonds and so on [114, 115, 148–150]. For example, Chen et al. have reported a thermo-sensitive hydrogel co-loaded DOX/IL-2/IFN-γ, which showed improved therapeutic efficacy B16F10 melanoma tumor by enhancing tumor cell apoptosis and increasing proliferation of the CD3^+^/CD4^+^ T cells and CD3^+^/CD8^+^ T cells [151]. Special hydrogel systems may have the ability for immune-stimulating. For example, Yang et al. have reported a melittin-RADA_32_ hydrogel-loaded DOX (MRD) for chemoimmunotherapy through active regulation of TMEs [152]. The melittin-RADA_32_ peptide, denoted as MR peptide, was a building block of the peptide hydrogel. Melittin is a cationic peptide derived from bee venom with the sequence GIGAVLKVLTTGLPALISWIKRKRQQ. The sequence of melittin-RADA_32_ was RADARADARADARADARADARADARADA-RADA-GG-GIGAVLKVLTTGLP-ALISWIKRKRQQ-NH2, in which melittin is linked to RADA_32_ through a GG linker. MRD showed enhanced killing effect to melanoma tumors by controlling drug release, regulating innate immune cells and depleting M2-type TAMs (Fig. 8).

### Biomimetic NPs

Biomimetic NPs have been designed to mimic natural organisms/structures through coating or mixing biocompatibility materials, which could camouflage of NPs as autologous components to escape the clearance of the immune system. Biomimetic NPs have the morphology, surface properties and size of natural structures (such as red blood cells, exosomes), which have enhanced targeting ability to deliver drugs to target cells or tissues, good biocompatibility, improved treatment efficiency and reduced side effects.

Proteins, such as albumin [153], high-density lipoproteins (HDL) [116], low-density lipoproteins (SDL), transferrin family proteins and their-derived proteins [154] have been applied to biomimetic NPs for chemoimmunotherapy. For example, HLD are involved in cholesterol and molecule transport, which could target specific cells. Anna Schwendeman et al. reported an HDL-mimicking nanodiscs loaded with CpG and DTX (DTX-sHDL-CpG) against glioblastoma multiforme (GBM). DTX-sHDL-CpG showed tumor inhibition and long-term survival in GBM tumor-bearing mice when combined with radiation [116]. The sHDL nanodisc was an effective NDDS for chemoimmunotherapy.

**Fig. 9:**
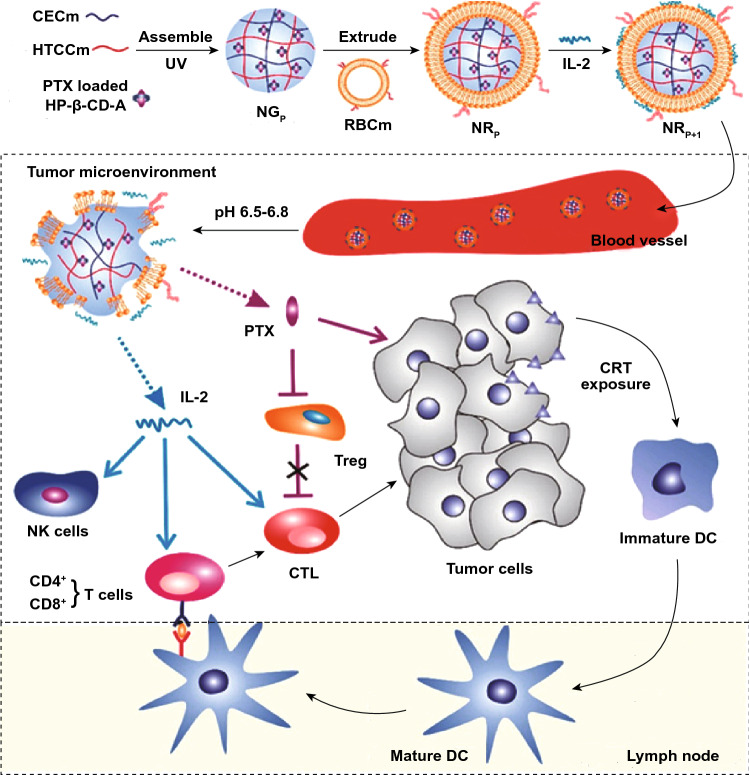
Schematic illustration NR_P+I_ for chemoimmunotherapy. Reproduced with permission from Ref. [118]. Copyright 2017 American Chemical Society

Cell membrane biomimetic NPs are mainly composed of cell membrane coating functional NPs. The proteins on the cell membranes derived from different cells still retain bioactive, thus giving them the ability to immune escape, prolonged blood circulation time and tumor targeting [155, 156]. Currently, cell membranes of biomimetic NPs mainly include erythrocyte, leukocytes, platelets, neutrophils, macrophages, T lymphocytes, stem cells and tumor cells [157–159]. Different cell membranes make biomimetic NPs have different functions in cancer therapy. Erythrocyte membrane biomimetic NPs could improve biocompatibility and biodegradability and prolong blood circulation [118]. For example, Zhiping Zhang and his group have developed erythrocyte membrane-coated nanogels (NR_P+I_) for PTX and IL-2 co-delivery and controlled release in TME (Fig. 9) [118]. The inner core nanogels were consisted of two opposite charged chitosan derivatives and 2-hydroxypropyl-β-cyclodextrin (HP-β-CD), which was for PTX loading and controlled release. The pH-responsive capability to acidic TME could be precisely controlled by adjusting the formulation of nanogel. The erythrocyte membrane was further coated on the nanogel for the delivery of IL-2. PTX may be controllable and pH-responsive released with the help of HP-β-CD and chitosan in TME. After losing the support of inner core, the membrane could be disintegrated to constantly release IL-2 into TME. NR_P+I_ showed enhanced antitumor activity with increased antitumor immunity and improved drug penetration. Tumor cell membrane biomimetic NPs have homologous targeting and homology adhesion, which enable its specific recognition and aggregation in tumor tissue [117]. Lymphocytes membrane biomimetic NPs could retain the ability to migrate to tumor sites and prolong circulation time in the body [160].

## Conclusion

The clinical and preclinical results uphold the rationality for the combination of chemotherapy and immunotherapy. Combining immunotherapy agents with chemotherapeutic drugs has great potentials as long-term maintenance therapy for cancer to maximize the synergistic antitumor effects. When designing a rational prescription for combinational chemoimmunotherapy in clinics, the administration dosages, intervals as well as cycles should be strategically considered to avoid iRAE effectively. NDDS provides a favorable platform in promoting the efficacy of chemoimmunotherapy, and numerous multifunctional NDDS have been designed up to now. These NDDS designed for chemoimmunotherapy showed many advantages, including increased solubility and bioavailability of both chemotherapy drugs and immunotherapy agents, prolonged circulation time in vivo, increased the accumulation of therapeutic agents in tumor site by specific-targeting and improved pharmacokinetics behaviors in vivo, thus significantly enhancing the therapeutic effects even at low-dose chemotherapeutic agents and reducing the side effects.

Despite NDDS-exhibited superiority in interrupting tumor immune balance, eliminating tumors and inhibiting metastasis, increased accumulation of immunotherapy agents at the tumor site might induce immunogenicity or autoimmune diseases and increase the occurrence of iRAE. Furthermore, chemotherapeutic agents and immunotherapy agents usually have different target cells, co-encapsulation strategies are difficult to achieve separate delivery to different cells in tumor tissue, which may unconsciously increase the off-target effect. Timing and quantitative controlled release of different drugs with precision targeting to different cells provide a new direction for the improvement in cancer chemoimmunotherapy or even immune-related multimodal-therapy. Otherwise, the clinical translation of NDDS are still facing significant obstacles due to complex processes, unavoidable drug leakage, undefined safety in excipients and undesired stability etc. In view of this, NDDS with simple prescription, mature preparation process and good biocompatibility are urgently pursued for higher clinical translation prospects. In a word, the combinational chemoimmunotherapy still has a long way to conquer in cancer treatment, and NDDS may play a crucial role in exerting their unique advantages.
